# FusionAtt: Deep Fusional Attention Networks for Multi-Channel Biomedical Signals

**DOI:** 10.3390/s19112429

**Published:** 2019-05-28

**Authors:** Ye Yuan, Kebin Jia

**Affiliations:** 1 College of Information and Communication Engineering, Beijing University of Technology, Beijing 100124, China; yuanye91@emails.bjut.edu.cn; 2 Beijing Key Laboratory of Computational Intelligence and Intelligent System, Beijing University of Technology, Beijing 100124, China

**Keywords:** attention mechanism, deep learning, biomedical signals, feature representation

## Abstract

Recently, pervasive sensing technologies have been widely applied to comprehensive patient monitoring in order to improve clinical treatment. Various types of biomedical signals collected by different sensing channels provide different aspects of patient health information. However, due to the uncertainty and variability in clinical observation, not all the channels are relevant and important to the target task. Thus, in order to extract informative representations from multi-channel biosignals, channel awareness has become a key enabler for deep learning in biosignal processing and has attracted increasing research interest in health informatics. Towards this end, we propose FusionAtt—a deep fusional attention network that can learn channel-aware representations of multi-channel biosignals, while preserving complex correlations among all the channels. FusionAtt is able to dynamically quantify the importance of each biomedical channel, and relies on more informative ones to enhance feature representation in an end-to-end manner. We empirically evaluated FusionAtt in two clinical tasks: multi-channel seizure detection and multivariate sleep stage classification. Experimental results showed that FusionAtt consistently outperformed the state-of-the-art models in four different evaluation measurements, demonstrating the effectiveness of the proposed fusional attention mechanism.

## 1. Introduction

With the broad adoption of pervasive sensors, comprehensive patient monitoring becomes feasible for a wide range of medical applications [1]. Various types of biomedical signals collected by different sensing channels (i.e., multi-channel biosignals) provide abundant physiological information and reflect different aspects of patients’ health condition. For instance, multi-channel scalp electroencephalogram (EEG) measures electrical activity in different brain areas, and each of these channels can be regarded as a sensing source providing unique brain information. It is applicable to several clinical tasks, including schizophrenia diagnosis [2], emotion recognition [3], and epileptic seizure detection [4]. In order to learn meaningful representations for the multi-channel biosignals, significant efforts were recently made to explore feature extraction techniques using deep neural networks [5,6]. These deep learning approaches are designed to capture abstract characteristics of signal patterns among all the biomedical channels, referred to as multi-view learning. However, not all the channels (or views) are relevant and important to the target task due to the uncertainty and variability in clinical observation (e.g., different brain regions are often involved in different types of brain activity) [4]. The irrelevance and redundant raw features may influence the model performance. Intuitively, an ideal deep learning approach should be able to distinguish the task-related importance among different channels and rely on more informative ones to enhance feature representation. Thus, channel awareness has become a key enabler for deep learning in biosignal processing, and has recently attracted increasing research interest in health informatics.

To address the channel awareness issue, several researchers have attempted to combine deep learning with a channel selection module, regarded as a multi-stage model, to mitigate the influence of irrelevance and redundancy in the raw feature space [4,7,8]. It has been validated that such deep-learning-based channel selection methods can improve the performance in modeling multi-channel biomedical data. However, these methods adopt the same selection strategy: only the critical channels are determined as the input to train the model, while the rest of the channels are one-off eliminated. Utilizing such a hard channel selection procedure ignores the detailed task-related information among channels over different situations. Take EEG seizure detection as an example: subjects have epileptic seizures to different extents (i.e., different brain regions), and hence the importance of each channel varies significantly across individuals. Moreover, since the existing deep-learning-based models extract features and select channels separately (i.e., multi-stage training), they do not consistently make all the functional modules work together, rendering it a challenging task to develop a unified channel-aware deep learning model.

To this end, we propose FusionAtt, a deep fusional attention network, to extract channel-aware representative features from multi-channel biosignals. We developed a new fusional attention layer that adopts a fusion gate to fully incorporate multi-view information, in order to dynamically measure the contribution of each biomedical channel. A multi-view convolutional encoding layer combined with convolutional neural networks (CNNs) [9] is also adopted to train a unified deep learning model. Experimental results conducted on different clinical datasets demonstrate that FusionAtt consistently obtained better performance than nine biosignal feature learning baselines in terms of four evaluation metrics. We summarize our main contributions as follows:

- We propose FusionAtt, a unified fusional attention neural network combined with multi-view convolutional encoder, designed to learn channel-aware representations of multi-channel biosignals.
- FusionAtt dynamically quantifies the importance of each biomedical channel by gated fusion, and relies on more informative ones to enhance feature representation, without prior expert knowledge.
- We empirically show that FusionAtt consistently achieved the best performance compared with nine biosignal feature learning baselines on two clinical datasets, demonstrating the effectiveness of the proposed fusional attention mechanism.

The rest of the paper is organized as follows: We first review the related work in the next section. In Section 3, we present the details of the proposed FusionAtt model. The experimental results are then discussed in Section 4. Finally, we conclude this work in Section 5.

## 2. Related Work

In this section, we summarize the literature related to our work in the following two categories: deep learning for multi-channel biosignals, and attention-based neural networks in clinical diagnosis.

### 2.1. Deep Learning for Multi-Channel Biosignals

Learning deep representations of biomedical data is crucial in the healthcare domain. The existing studies on deep learning for multi-channel biosignals are diverse due to the wide range of medical applications. Deep belief networks (DBNs) have been widely adopted to learn inherent representations of polysomnography (PSG) signals to classify sleep stages [10,11]. To capture temporal patterns from multi-channel waveform data, context learning was employed for emotion recognition [3] and seizure detection [12,13]. CNN-based approaches were also proposed to fuse information from different sensing sources into a high-order feature space [14,15]. Compared with these models that utilize parameter sharing architecture to extract joint features among channels, we integrate features explicitly according to the relative significance of each biomedical channel.

More recently, several researchers have attempted to combine deep neural networks with a channel selection module in order to mitigate the influence of irrelevance and redundancy in the raw feature space. Yuan et al. [4] considered the response energy of stacked autoencoders (SAEs) to jointly determine critical EEG channels. Li et al. [7] and Jia et al. [8] proposed different DBN-based channel selection modules to recognize affective state from multi-channel biosignal data. However, these advanced methods, though yielding reasonably good performance, adopt hard channel selection strategies and are not unified models. In contrast, we propose the fusional attention mechanism to dynamically select and fuse channel information in an end-to-end manner.

### 2.2. Attention-Based Neural Networks in Clinical Diagnosis

Recently, attention mechanisms have attracted increasing research interest in clinical diagnosis, due to their strong ability of feature extraction and model interpretability. Choi et al. [16] and Ma et al. [17] adopted attention mechanisms to explain medical codes (e.g., procedure, diagnosis, and medication codes) from electronic health records (EHRs). Yuan et al. [18] first exploited an attention mechanism based on multi-view learning (i.e., ChannelAtt) to achieve soft channel selection for EEG seizure detection. In general, there are two differences between ChannelAtt and our proposed FusionAtt model. First, ChannelAtt uses local information and concatenated information to assign attention energy, referred to as local attention and global attention, respectively. In our FusionAtt model, we adopt a gated function to better fuse the multi-view information for the attention energy assignment. Second, the multi-view representation module in ChannelAtt is based on a SAE, a fully-connected neural network, while ours is based on a CNN, a locally-connected neural network, which can better extract deep features from multi-channel biosignals.

## 3. Methodology

**Figure 1:**
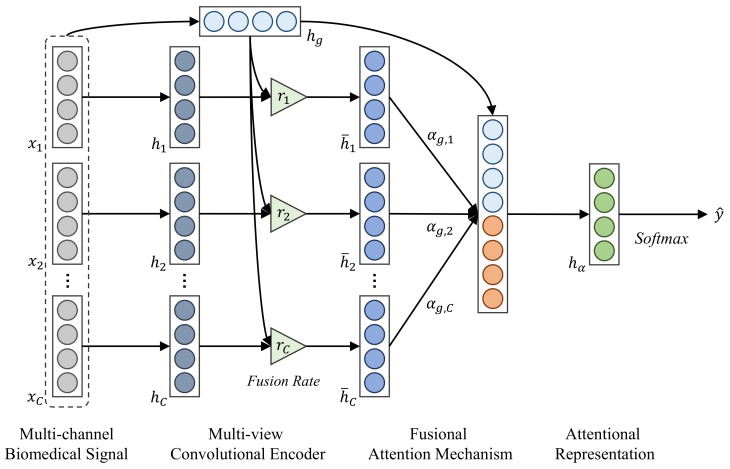
Architecture of the proposed FusionAtt model.

In this section we present the technical details of our FusionAtt model with multi-channel biosignal inputs. The architecture of FusionAtt is illustrated in Figure 1. In the following subsections, we detail the main components of our FusionAtt model.

### 3.1. Multi-View Convolutional Encoder

In order to learn deep representations of multi-channel waveform data, simply concatenating the raw inputs of all the channels may not be enough to preserve the unique characteristics of each channel. Inspired by the rapid development of multi-view deep learning techniques [4,15,19], we propose the utilization of two convolutional feature encoders (i.e., channel-encoder and global-encoder) to extract abstract features from channel-specific and global views, respectively.

Formally, we assume that the input biosignal fragments consist of *C* channels, denoted as $X=\{x_{1},\cdots ,x_{i},\cdots ,x_{C}\}$. Given the input vector of the *i*-th channel, denoted as $x_{i}\in R^{n}$, a channel-view representation $h_{i}\in R^{p}$ can be obtained using the channel-encoder (i.e., $Encoder_{c}$), as follows:

(1) $$h_{i}=Encoder_{c}\left(x_{i};\theta_{c}\right),$$

where $\theta_{c}$ is the learnable parameter set in $Encoder_{c}$. Similarly, we can calculate a global-view representation $h_{g}\in R^{p}$ through the global-encoder (i.e., $Encoder_{g}$), as follows:

(2) $$h_{g}=Encoder_{g}\left(x_{1:C};\theta_{g}\right),$$

where $\theta_{g}$ is the learnable parameter set $Encoder_{g}$. Generally speaking, both $Encoder_{c}$ and $Encoder_{g}$ can be parameterized by different deep learning methods designed for feature extraction.

In our model, we construct the multi-view convolutional encoder by stacking several multi-kernel CNN cells consisting of convolutional, nonlinear, and pooling layers. Specifically, regarding our two feature encoders, the latent multi-view representations of the *k*-th feature map, denoted as ${\tilde{h}}_{i}^{\left(k\right)}$ and ${\tilde{h}}_{g}^{\left(k\right)}$, can be obtained as follows:

(3) $${\tilde{h}}_{i}^{\left(k\right)}=f\left(x_{i}*W_{c}^{\left(k\right)}+b_{c}^{\left(k\right)}\right),$$

(4) $${\tilde{h}}_{g}^{\left(k\right)}=f\left(\sum_{i=1}^{C}x_{i}*W_{g}^{\left(k\right)}+b_{g}^{\left(k\right)}\right),$$

where $\theta_{g}^{\left(k\right)}=\left\{W_{c}^{\left(k\right)},b_{c}^{\left(k\right)}\right\}$ and $\theta_{c}^{\left(k\right)}=\left\{W_{g}^{\left(k\right)},b_{g}^{\left(k\right)}\right\}$ are the learnable parameters. Subsequently, we flatten all the features extracted by different kernels and derive the global-view and channel-view representations, that is, $h_{i}$ and $h_{g}$, respectively. Note that the dimension of ${\tilde{h}}_{i}^{\left(k\right)}$ and ${\tilde{h}}_{g}^{\left(k\right)}$ relies on the structure configuration of the multi-view convolutional encoder, which is given in Section 4.3.

### 3.2. Fusional Attention Mechanism

In order to dynamically qualify the contribution of each biomedical channel, we propose the fusional attention mechanism, which incorporates a gated function for the final task. Specifically, the fusion gate $r_{i}\in R$ can be calculated considering both the global-view and channel-view representations, defined as:

(5) $$r_{i}=\sigma \left(W_{rg}^{⊤}h_{g}+W_{rc}^{⊤}h_{i}+b_{rc}\right),$$

where $\theta_{a}^{\left(1\right)}=\left\{W_{rg},W_{rc}\in R^{p},b_{rc}\in R\right\}$ denotes the learnable parameter set. Here in Equation (5), we rescale $r_{i}$ into the range of [0,1] by adopting the sigmoid function σ(·), in order to control the flow of multi-view information through the neural networks. We then integrate the information of the global-view and its own channel-view representations according to the fusion gate $r_{i}$, defined as:

(6) $${\bar{h}}_{i}=\left(1-r_{i}\right)⊙h_{g}+r_{i}⊙h_{i},$$

where ⊙ is the element-wise multiplication operator. According to Equation (6), the fusion gate $r_{i}$ is able to learn how much information carried by each encoder is relevant to keep or forget during end-to-end training. If $r_{i}=1$, then ${\bar{h}}_{i}=h_{i}$. This means that only channel-view information is passed. if $r_{i}=0$, then ${\bar{h}}_{i}=h_{g}$. This means that only global information is passed. We use the gated unit to derive a more representative integrated feature vector, that is, ${\bar{h}}_{i}\in R^{p}$, as the input of the attention energy assignment function.

The attention energy $e_{g,i}$ of the *i*-th channel can be further assigned based on the integrated feature vector ${\bar{h}}_{i}$, as follows:

(7) $$e_{g,i}=W_{e}^{⊤}{\bar{h}}_{i}+b_{e},$$

where $\theta_{a}^{\left(2\right)}=\left\{W_{e}\in R^{p},b_{e}\in R\right\}$ is the learnable parameter set. Given all the attention energy values, the contribution score vector $\alpha_{g}\in R^{C}$ can be normalized using softmax function, as follows:

(8) $$\alpha_{g}=Softmax\left(\left[e_{g,1},\cdots ,e_{g,i},\cdots ,e_{g,C}\right]\right).$$

Intuitively, if the contribution score $\alpha_{g,i}$ of the *i*-th channel is large, the information of the *i*-th channel is high related to the corresponding task label. Subsequently, we use weighted aggregation to compute a context vector $c_{g}\in R^{p}$ based on the integrated features ${\bar{h}}_{i}\left(1\leq i\leq C\right)$ and the contribution score vector $\alpha_{g}$, as follows:

(9) $$c_{g}=\sum_{i=1}^{C}\alpha_{g,i}⊙{\bar{h}}_{i}.$$

In this way, our model is able to effectively incorporate the multi-view information carried by both feature views, and hence fuse representative features from multi-channel biosignals.

### 3.3. Unified Training Procedure

To train our FusionAtt model in an end-to-end manner, we combine the context vector with the global-view vector to derive an attentional representation $h_{\alpha }\in R^{r}$, defined as:

(10) $$h_{\alpha }=f\left(W_{h}\left[c_{g}⊕h_{g}\right]+b_{h}\right),$$

where ⊕ is the concatenation operator, and $W_{h}\in R^{r\times 2p}$ and $b_{h}\in R^{r}$ are the learnable parameters. Finally, a softmax layer is applied to produce the classification task, as follows:

(11) $$\hat{y}=Softmax\left(W_{s}h_{\alpha }+b_{s}\right),$$

where $W_{s}\in R^{|class|\times r}$ and $b_{s}\in R^{\left|class\right|}$ denote the learnable parameters. Here we employ cross-entropy as the classification loss. Given *M* training samples ${\left\{\left(X^{\left(m\right)},y^{\left(m\right)}\right)\right\}}_{m=1}^{M}$, the cost function of our unified FusionAtt network in terms of the learnable parameter set $\Theta =\{\theta_{c},\theta_{g},\theta_{a},W_{h,s},b_{h,s}\}$, is defined as:

(12) $$\begin{array}{c} J_{FusionAtt}\left(X^{\left(1\right)},\cdots ,X^{\left(m\right)},\cdots ,X^{\left(M\right)};\Theta \right)\\ =\;-\frac{1}{M}\sum_{m=1}^{M}\left[y^{\left(m\right)}\log{\hat{y}}^{\left(m\right)}+\left(1-y^{\left(m\right)}\right)\log\left(1-{\hat{y}}^{\left(m\right)}\right)\right]. \end{array}$$

## 4. Experiments

In this section, we evaluate FusionAtt using two benchmark clinical tasks: multi-channel EEG seizure detection and multivariate PSG sleep stage classification. We first introduce the two datasets, then describe the baselines and implementation details. We finally present the quantitative results and analyze the learned contribution scores through a clinical case study.

### 4.1. Datasets

*CHB-MIT.* We performed the task of multi-channel EEG seizure detection using the public CHB-MIT dataset provided by the Children’s Hospital Boston [20]. This dataset contains 23-channel 256 Hz EEG signals. All the seizures are manually labeled by medical experts. Following the segmentation experience [21], we set both window length and step length as 1 second, and finally generated 252,862 input vectors from all 23 subjects.

*UCD.* We conducted experiments for the multivariate PSG sleep stage classification task based on the UCD dataset collected from St. Vincent’s University Hospital and University College Dublin [22]. This dataset contains 14-channel overnight PSG data, consisting of 128 Hz EEG, 64 Hz electromyography (EMG), and other types of biosignals. We generated 287,840 input vectors from all 25 subjects, and each 30-second fragment is labeled as being in one of the five sleep stages.

### 4.2. Baseline Approaches

We compared FusionAtt with the following nine existing biosignal feature learning baselines:

*Support vector machine (SVM)* [23]. SVM is a classic machine learning method. Here we adopted a one-vs-all SVM for the multi-class classification. To avoid the curse of dimensionality, we used principal component analysis (PCA) [24] to select the top-*r* related components from all channels as features before training the SVM, namely, PSVM.

*SAE* [25]. SAE is a widely adopted deep learning method for biosignal feature learning. For the sake of fairness, we incorporated global and multi-view strategies to extend the SAE model, referred to as GSAE and MSAE, respectively.

*CNN* [9]. The CNN is another commonly used deep learning method in biosignal processing. Similarity, we performed the same process as with SAE (i.e., GCNN and MCNN).

*CtxFusionEEG* [13]. CtxFusionEEG is a multi-stage feature learning model that focuses on using context learning to detect temporal patterns from epilepsy EEG signals. This method derives fusional representations of multi-channel biosignals by incorporating both deep learning and handcrafted engineering.

*mSSDA* [4]. mSSDA is an SAE-based multi-view deep-learning variant for multi-channel biosignals. This method determines channels by adopting a hard selection procedure after the deep feature extraction.

*ChannelAtt* [18]. ChannelAtt adopts fully connected multi-view learning to soft-select critical channels from multi-channel biosignals. According to the original model, both the local and global attention mechanisms are included, referred to as ChannelAtt${}_{loc}$ and ChannelAtt${}_{glo}$, respectively.

### 4.3. Implementation Details

We implemented our proposed model with PyTorch. Regarding the evaluation procedure, 5-fold subject-independent cross-validation was adopted, and we report the average test results for performance comparisons. The ratio of training, validation, and test sets was 0.7:0.1:0.2. Furthermore, we utilized short-time Fourier transform (STFT) for data preprocessing. Adadelta was adopted for the training process to optimize the cost function in terms of the learnable parameters. We also used weight decay with a 0.001L2 penalty coefficient, 0.95 momentum, and 0.5 dropout rate to train all the deep learning methods. Table 1 lists the structure configuration of the multi-view convolutional encoder in FusionAtt, and we set p=128 and r=128 for our model and baselines.

To quantify the performance, both Accuracy and F1-score were adopted for evaluation. The area-under-the-curve of precision–recall (AUC-PR) and receiver operator characteristic (AUC-ROC) were also utilized to evaluate each approach. Note that we used the Macro metric for the multi-class classification task.

### 4.4. Performance on Clinical Tasks

We investigated the effectiveness of our proposed FusionAtt model compared to the baseline methods in different biomedical tasks. Table 2 reports the experimental results of all the aforementioned approaches on all the benchmark datasets. We can see that our FusionAtt model achieved the best performance compared with the corresponding baselines utilizing different encoder methods on both datasets.

Given the results of the baselines, the traditional classification method PSVM outperformed the SAE-based models in both tasks. This demonstrates the effectiveness of the handcrafted PCA features where SVM could find a relatively clear hyper-lane to separate classes in the vector space. Among all the deep learning baselines, we observed that the multi-view-based methods obtained better results than the globally based methods. This illustrates the advantage of multi-view representation, in which the meaningful features are effectively extracted from multi-channel biosignal data. The improvement of the CNN-based methods compared with the SAE-based methods demonstrates that all the deep learning models take advantage of the CNN feature encoder. The reason is that the spatial information of channels is retained and the classifier can hence capture more detailed information from multi-channel biosignals. Since mSSDA employs the hard channel selection procedure, all the evaluation measurements increase compared with the SAE-based methods. This suggests the benefit of channel selection on multi-channel biosignals to capture critical information. CtxFusionEEG performed on par with mSSDA in terms of accuracy, but achieved a much worse F1-score than mSSDA, even the other deep learning baselines. This means that adopting multi-stage training would not consistently make all the functional modules work together to yield good results across different classes.

**Figure 2:**
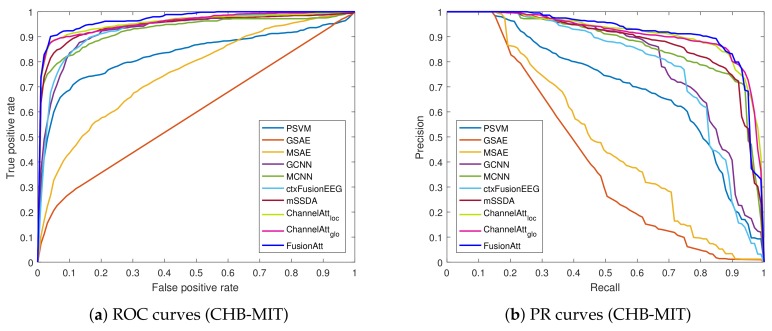
Receiver operating characteristic (ROC) and precision–recall (PR) curves of the proposed method and the baselines on the CHB-MIT dataset. (**a**) ROC curves; (**b**) PR curves.

**Figure 3:**
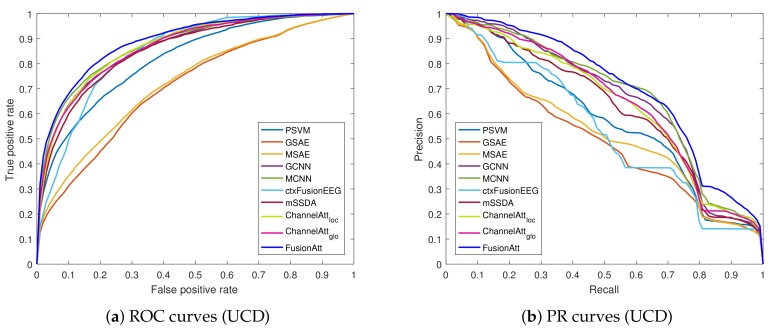
ROC and PR curves of the proposed method and the baselines on the UCD dataset. (**a**) ROC curves; (**b**) PR curves.

From the results of the attention-based models, the comparisons between ChannelAtt${}_{loc}$ and ChannelAtt${}_{glo}$ indicate that the concatenated information captured by the global attention mechanism is helpful for feature extraction. Our proposed FusionAtt model performed better than all the baselines, due to the effective integration of the multi-view representation and fusional attention mechanism. Moreover, Figure 2 and Figure 3 illustrate the ROC and PR curves of all the test folds on the CHB-MIT and UCD datasets, respectively. We can see that FusionAtt achieved the best performance in terms of the AUC-ROC and AUC-PR. This means that FusionAtt was able to focus on providing complementary information toward each view representations, and hence could help to learn more informative features from multi-channel biosignal data. According to the overall analysis on both datasets, we conclude that the fusional attention mechanism is key to identifying critical patterns from biomedical signals, and the attentional representation learned by FusionAtt can improve the performance for different biomedical tasks in healthcare.

### 4.5. Sensitivity Analysis

In this subsection, we discuss the effects of various hyper-parameter choices in FusionAtt, including the dimensionality of multi-view representation *p* and the size of attentional representation *r*. Specifically, we plot the accuracy results of our proposed FusionAtt model and the two ChannelAtt variants under different hyper-parameter settings on both datasets. Here we use the hyper-parameter setting mentioned in Section 4.3 as the basic configuration, and vary one from 32 to 512 while keeping others fixed to the basic configuration.

**Figure 4:**
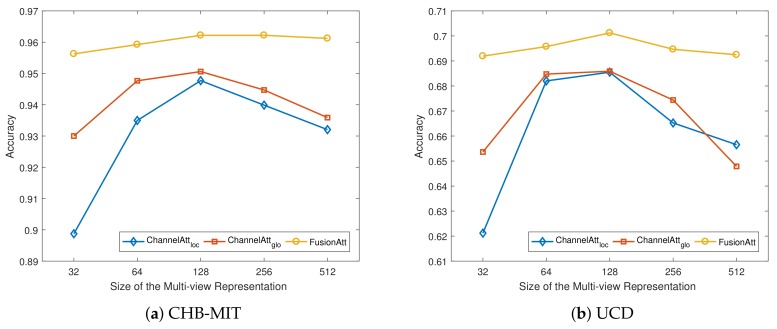
Performance variations with different dimensions of multi-view representation. (**a**) Sensitivity analysis on the CHB-MIT dataset; and (**b**) Sensitivity analysis on the UCD dataset.

**Figure 5:**
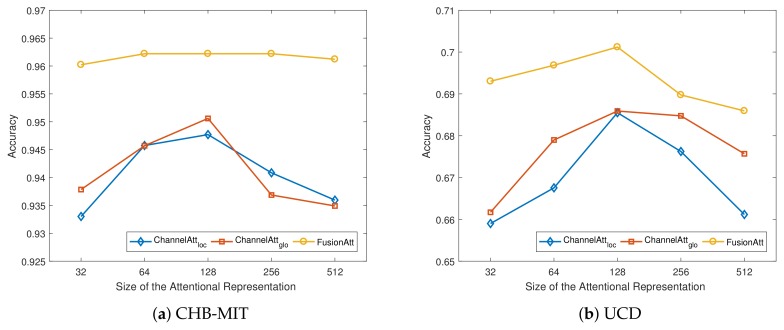
Performance variations with different dimensions of attentional representation. (**a**) Sensitivity analysis on the CHB-MIT dataset; (**b**) Sensitivity analysis on the UCD dataset.

Figure 4 illustrates the variation of accuracy under different sizes of multi-view representation *p* using different feature encoders on the two datasets. From the figures, we observe that when the dimension was small, all the models resulted in limited accuracy performance, demonstrating that a smaller size of *p* led to worse capture of the multi-channel information. When we increased *p*, the model performance increased rapidly. However, when *p* was too large, the performance decreased slightly. This shows that properly setting the size of multi-view representation can help to generate robust features to improve the performance, while excessive hidden neurons negatively impact the model performance. Similarity, as shown in Figure 5, we can see the same trends of the influence under different sizes of attentional representation *r*. Among all the figures, regarding the feature encoders, the proposed FusionAtt model consistently beat the two ChannelAtt models. This resulted from the fact that the multi-view convolutional encoder has strong model generalization capability. Moreover, the FusionAtt model was less sensitive to different choices of hyper-parameters than the other models. This demonstrates that the fusion gate in Equation (5) enables FusionAtt to dynamically fuse features from multiple views, deriving more comprehensive features from multi-channel biosignals.

### 4.6. Case Study

**Figure 6:**
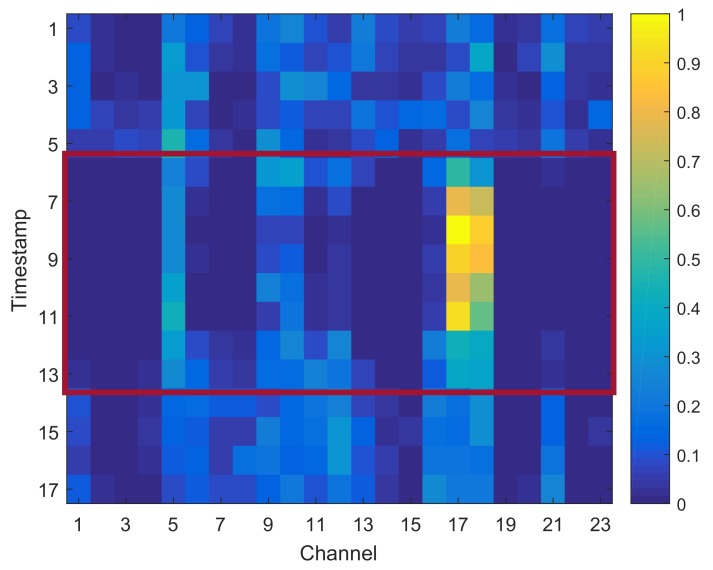
Multi-channel electroencephalogram (EEG) seizure detection of a patient in the case study.

In this subsection, we discuss the learned contribution scores in FusionAtt to justify the benefit of adopting the fusional attention mechanism in clinical settings. Figure 6 presents a clinical case study of multi-channel EEG seizure detection on the CHB-MIT dataset where a subject suffered epileptic seizure from the 6-th to 13-th timestamps. We display the mean scores of every 5 fragments and mark the max value among all the channels within each timestamp for clear visualization. Note that according to the EEG seizure detection results discussed in Section 4.4, the contribution scores learned from all of the channel-aware attention variants were similar, due to the similar attention learning scenarios.

From Figure 6, we can observe that the learned contribution scores of all the channels were different within different timestamps. Analyzing the timestamps that belong to the non-seizure stage, the distribution of contribution scores was relatively uniform. Therefore, no seizure pattern was recognized, and hence our model paid equal attention to all the EEG channels in detecting seizure onset. Regarding the timestamps in the seizure stage, we can observe that the 17-th and 18-th channels were the two most active ones that significantly contributed to the final task of seizure detection. This indicates that the seizure onset was highly related to the brain regions measured by these two channels. According to the International 10–20 system employed in this dataset [20], the seizure onset in this case was located in the central region of the brain, which conforms to the channels selected by our model. To sum up, the case study justifies the ability of our proposed FusionAtt model to learn interpretable contribution scores with clinical meanings. According to Table 2, the meaningful representations support the effectiveness of FusionAtt for good performance in seizure detection.

## 5. Conclusions

In this paper, we present deep fusional attention networks, namely FusionAtt, to learn deep representations of multi-channel biosignals for clinical tasks. The proposed FusionAtt is a unified deep learning framework that fuses gated attentional features by capturing dependencies among biomedical channels based on multi-view convolutional encoding. Experiments on two benchmark clinical tasks showed that FusionAtt was able to efficiently fuse channel-aware information from multi-channel biosignals. The case study intuitively showed how FusionAtt was able to interpret influential clinical observations by analyzing the learned contribution scores of biomedical channels.

Our future work will include the incorporation of different deep-learning architectures for feature encoding, in addition to investigating the performance of FusionAtt on larger datasets. In addition, FusionAtt can be extended to other task-oriented applications with similar data structure where the channel awareness is still a major challenge.

## Figures and Tables

**Table 1:** Structure configuration of the multi-view convolutional encoder. ReLU: rectified linear unit.

Cell No.	Conv	Non-linear	Pooling
$Encoder_{c1}$	(8,16,32,64)×8	ReLU	6
$Encoder_{c2}$	(3,5)×16	ReLU	3
$Encoder_{c3}$	(3,5)×16	ReLU	3
$Encoder_{g1}$	(1×8,1×16,1×32,1×64)×8	ReLU	1×6
$Encoder_{g2}$	(3×3,5×5)×16	ReLU	1×3
$Encoder_{g3}$	(3×3,5×5)×16	ReLU	C×3

**Table 2:** Performance comparisons on two clinical biosignal datasets. GCNN: global extension of convolutional neural network (CNN); MCNN: multi-view extension of CNN; GSAE: global extension of stacked autoencoder (SAE); MSAE: multi-view extension of SAE.

Method	Seizure Detection (CHB-MIT)				Sleep Stage Classification (UCD)			
	AUC-ROC	AUC-PR	F1-Score	Accuracy	AUC-ROC	AUC-PR	F1-Score	Accuracy
PSVM	0.8291	0.7021	0.6421	0.8768	0.8177	0.5764	0.5204	0.6193
GSAE	0.5934	0.4180	0.0668	0.7987	0.7068	0.4965	0.2760	0.4917
MSAE	0.7529	0.4937	0.1479	0.8013	0.7213	0.5224	0.3542	0.5262
GCNN	0.9255	0.8054	0.7506	0.8849	0.8655	0.6589	0.5042	0.6270
MCNN	0.9263	0.8702	0.7959	0.9088	0.8732	0.6725	0.5925	0.6590
CtxFusionEEG	0.9287	0.7833	0.7202	0.9025	0.8483	0.5330	0.4680	0.6688
mSSDA	0.9450	0.8801	0.8186	0.9364	0.8544	0.6208	0.5969	0.6741
ChannelAtt${}_{loc}$	0.9554	0.9134	0.8625	0.9477	0.8699	0.6370	0.5890	0.6855
ChannelAtt${}_{glo}$	0.9556	0.9119	0.8675	0.9506	0.8662	0.6458	0.6137	0.6859
FusionAtt	0.9701	0.9145	0.8953	0.9622	0.8894	0.7021	0.6637	0.7257
